# Hesperetin, a Citrus Flavonoid, Ameliorates Inflammatory Cytokine-Mediated Inhibition of Oligodendroglial Cell Morphological Differentiation

**DOI:** 10.3390/neurolint14020039

**Published:** 2022-05-31

**Authors:** Satoshi Nishino, Yoko Fujiki, Takanari Sato, Yukino Kato, Remina Shirai, Hiroaki Oizumi, Masahiro Yamamoto, Katsuya Ohbuchi, Yuki Miyamoto, Kazushige Mizoguchi, Junji Yamauchi

**Affiliations:** 1 Laboratory of Molecular Neurology, Tokyo University of Pharmacy and Life Sciences, Hachioji, Tokyo 192-0392, Japan; satoshi-nishino@g.ecc.u-tokyo.ac.jp (S.N.); s177061@toyaku.ac.jp (Y.F.); s177030@toyaku.ac.jp (T.S.); s179026@toyaku.ac.jp (Y.K.); rshirai@toyaku.ac.jp (R.S.); miyamoto-y@ncchd.go.jp (Y.M.); 2 Tsumura Research Laboratories, Tsumura & Co., Inashiki, Ibaraki 200-1192, Japan; ooizumi_hiroaki@mail.tsumura.co.jp (H.O.); hirokoma@h.email.ne.jp (M.Y.); oobuchi_katsuya@mail.tsumura.co.jp (K.O.); mizoguchi_kazushige@mail.tsumura.co.jp (K.M.); 3 Laboratory of Molecular Pharmacology, National Research Institute for Child Health and Development, Setagaya, Tokyo 157-8535, Japan

**Keywords:** oligodendrocyte, differentiation, TNFα, IL-6, hesperetin, Akt kinase

## Abstract

Oligodendrocytes (oligodendroglial cells) are glial cells that wrap neuronal axons with their differentiated plasma membranes called myelin membranes. In the pathogenesis of inflammatory cytokine-related oligodendroglial cell and myelin diseases such as multiple sclerosis (MS), typical inflammatory cytokines tumor necrosis factor α (TNFα) and interleukin-6 (IL-6) are thought to contribute to the degeneration and/or progression of the degeneration of oligodendroglial cells and, in turn, the degeneration of naked neuronal cells in the central nervous system (CNS) tissues. Despite the known involvement of these inflammatory cytokines in disease progression, it has remained unclear whether and how TNFα or IL-6 affects the oligodendroglial cells themselves or indirectly. Here we show that TNFα or IL-6 directly inhibits morphological differentiation in FBD-102b cells, which are differentiation models of oligodendroglial cells. Their phenotype changes were supported by the decreased expression levels of oligodendroglial cell differentiation and myelin marker proteins. In addition, TNFα or IL-6 decreased phosphorylation levels of Akt kinase, whose upregulation has been associated with promoting oligodendroglial cell differentiation. Hesperetin, a flavonoid mainly contained in citrus fruit, is known to have neuroprotective effects. Hesperetin might also be able to resolve pre-illness conditions, including the irregulated secretion of cytokines, through diet. Notably, the addition of hesperetin into cells recovered TNFα- or IL-6-induced inhibition of differentiation, as supported by increased levels of marker protein expression and phosphorylation of Akt kinase. These results suggest that TNFα or IL-6 itself contributes to the inhibitory effects on the morphological differentiation of oligodendroglial cells, possibly providing information not only on their underlying pathological effects but also on flavonoids with potential therapeutic effects at the molecular and cellular levels.

## 1. Introduction

Oligodendrocytes (also called oligodendroglial cells) are glial cells that wrap around neuronal axons with their myelin sheaths as differentiated plasma membranes in the central nervous system (CNS) [1,2,3,4]. Once fully formed, these myelin sheaths not only allow fast electrical conduction but also protect axons from stresses such as physical insults [1,2,3,4,5,6]. Although the generation of these mature myelin sheaths by oligodendroglial cells has been studied, it remains unclear how oligodendroglial cells are affected in pathological states.

Inflammatory cytokines are often associated with oligodendroglial cell- and myelin-related diseases. For example, inflammatory cytokine-associated diseases such as multiple sclerosis (MS) are neurological disorders characterized by demyelination and neurodegeneration of the brain and spinal cord of the CNS. In these cases, immune cells themselves or immune systems, possibly acting through soluble factors such as cytokines or membrane-bound molecules, are thought to attack myelin sheaths and peel them off from myelinated nerves, causing a breakdown of communications between neuronal cells. Eventually, these diseases can lead to the deterioration or permanent damage of neuronal cells [7,8,9]. Although their pathogenesis is not completely understood, increasing evidence suggests that inflammatory cytokines such as tumor necrosis factor α (TNFα) and interleukin-6 (IL-6), which are secreted by immune cells or other cells, are probably related to triggering incomplete or defective oligodendroglial cell differentiation and demyelinating phenomena. TNFα and IL-6 secretion is thus likely to be associated with the onset and/or progression of these diseases, including MS [7,8,9,10]. All of these circumstances combined result in persistent demyelinating states and, in turn, neuronal degradation.

The answer to the question of whether treatment with an inflammatory cytokine TNFα or IL-6 directly or indirectly affects the morphology of oligodendroglial cells has been awaited. Herein we describe how TNFα or IL-6 affects oligodendroglial cell morphological differentiation in FBD-102b cells, the model cells of oligodendroglial cells that can undergo differentiation [11,12,13,14]. We also investigate the effects of hesperetin on cells treated with TNFα or IL-6. Hesperetin is a major flavonoid that is abundant in many citrus fruits and an aglycone of hesperidin [15,16]. Hesperetin has neuroprotective effects and may have health-promoting effects on the whole body [15,16]. It may thus be possible to treat pre-illness situations through diet, such as conditions caused by the abnormal regulation of cytokines.

## 2. Materials and Methods

### 2.1. Antibodies and Recombinant Proteins

Antibodies were purchased from the following companies. Rabbit polyclonal anti-proteolipid protein 1 (PLP1, Cat. No. HPA004128; IB, 1/1000) was purchased from Atlas Antibodies (Bromma, Sweden); mouse monoclonal anti-Sox10 (Cat. No. sc-365692; IB, 1/500) from Santa Cruz Biotechnology (Santa Cruz, CA, USA); mouse monoclonal anti-actin (Cat. No. M177-3; IB, 1/40,000) from MBL (Aichi, Japan); and rabbit polyclonal anti-Akt1 (Cat. No. C73H10; IB, 1/500) and mouse monoclonal anti-(pS473)Akt1 (Cat. No. D7F10; IB, 1/500), which recognizes the phosphorylation site needed for kinase activation, from Cell Signaling Technology (Danvers, MA, USA). The following secondary antibodies were purchased: anti-rabbit or mouse IgG F(ab’) conjugated with horseradish peroxidase (Cat. Nos. 458 or 330; IB, 1/5000) from MBL.

Recombinant mouse TNFα or IL-6 proteins (Cat Nos. 315-01A or 216-16) as well as IL-1α (Cat. No. 211-11A), IL-1β (Cat. No. 211-11B), or IL-11 (Cat. No. 220-11) were purchased from PeproTech, Inc. (Cranbury, NJ, USA).

### 2.2. Cell Culture and Differentiation

Mouse brain oligodendroglial FBD-102b cells were cultured on cell culture dishes in Dulbecco’s Modified Eagle’s Medium (DMEM)/F-12 mixed medium containing 10% heat-inactivated fetal bovine serum (FBS) and penicillin-streptomycin mixed solution (Nacalai Tesque, Kyoto, Japan) in 5% CO_2_ at 37 °C. FBD-102b cells were kindly provided by Dr. Y. Tomo-oka (Tokyo University of Science, Chiba, Japan and Riken, Saitama, Japan).

To induce differentiation, FBD-102b cells were cultured on polylysine-coated cell culture dishes (Greiner Bio-One, Oberösterreich, Austria) in culture medium without FBS for 0 to 3 days with 50 ng/mL of each cytokine or control vehicles in 5% CO_2_ at 37 °C. Cells were generally cultured in the presence of 10 μM of hesperetin (Nacalai Tesque) or control vehicles except for studies looking at hesperetin dosing. Cells with multiple processes from the cell bodies were identified as differentiated [11,12,13,14]. Cells with primary processes were classified as category 1; cells with secondary processes branched from primary processes were classified as category 2; and cells with third processes branched from secondary processes or with myelin membrane-like widespread membranes were classified as category 3 (in some cases, cells with myelin membrane-like widespread membranes were classified as category 4). Cells with the category 3 stage (in some cases, the category 4 stage) were generally considered the most differentiated cells. In contrast, category 1 was considered to be the phenotypes before differentiation whereas category 3 was considered to be the differentiated phenotypes. Category 2 corresponded to these intermediate phenotypes [11]. Cell morphologies were determined by microscopic systems equipped with i-NTER LENS (Micronet, Inc., Saitama, Japan).

### 2.3. Denatured Polyacrylamide Electrophoresis and Immunoblotting

Following the induction of differentiation for 2 days, cells were lysed in lysis buffer (50 mM HEPES-NaOH, pH 7.5, 150 mM NaCl, 20 mM MgCl_2_, 1 mM dithiothreitol, 1 mM phenylmethane sulfonylfluoride, 1 μg/mL leupeptin, 1 mM EDTA, 1 mM Na_3_VO_4_, 10 mM NaF, and 0.5% NP-40) to be collected by centrifuge as cell lysates. For denatured conditions, their supernatants were denatured sample buffer (Nacalai Tesque). The samples were separated on pre-made sodium dodecylsulfate–polyacrylamide gels (Nacalai Tesque). The electrophoretically separated proteins were transferred to PVDF membranes (Fujifilm, Tokyo, Japan) sandwiched between filter papers, blocked with Blocking One (Nacalai Tesque), and immunoblotted using primary antibodies, followed by peroxidase-conjugated secondary antibodies. The Canoscan LiDE400 image scanner (Canon, Tokyo, Japan)-captured, peroxidase-reactive bands on X-ray films (Fujifilm) were analyzed using UN-SCAN-IT software (Silk Scientific, Orem, UT, USA). We performed some sets of experiments in immunoblotting studies and quantified other immunoreactive bands with the control’s immunoreactive band as 100% with NIH ImageJ software (Bethesda, MD, USA).

### 2.4. Statistical Analyses

Values are the means ± standard deviation (SD) from separate experiments. Intergroup comparisons were made using the unpaired Student’s *t*-test using Excel (Microsoft, Redmond, WA, USA). A one-way analysis of variance (ANOVA) was followed by a Fisher’s protected least significant difference (PLSD) test as a post hoc comparison using StatPlus (AnalystSoft, Walnut, CA, USA). Differences were considered statistically significant when *p* < 0.05.

## 3. Results

### 3.1. TNFα or IL-6 Inhibits Differentiation Whose State Is Related to Akt Phosphorylation

**Figure 1:**
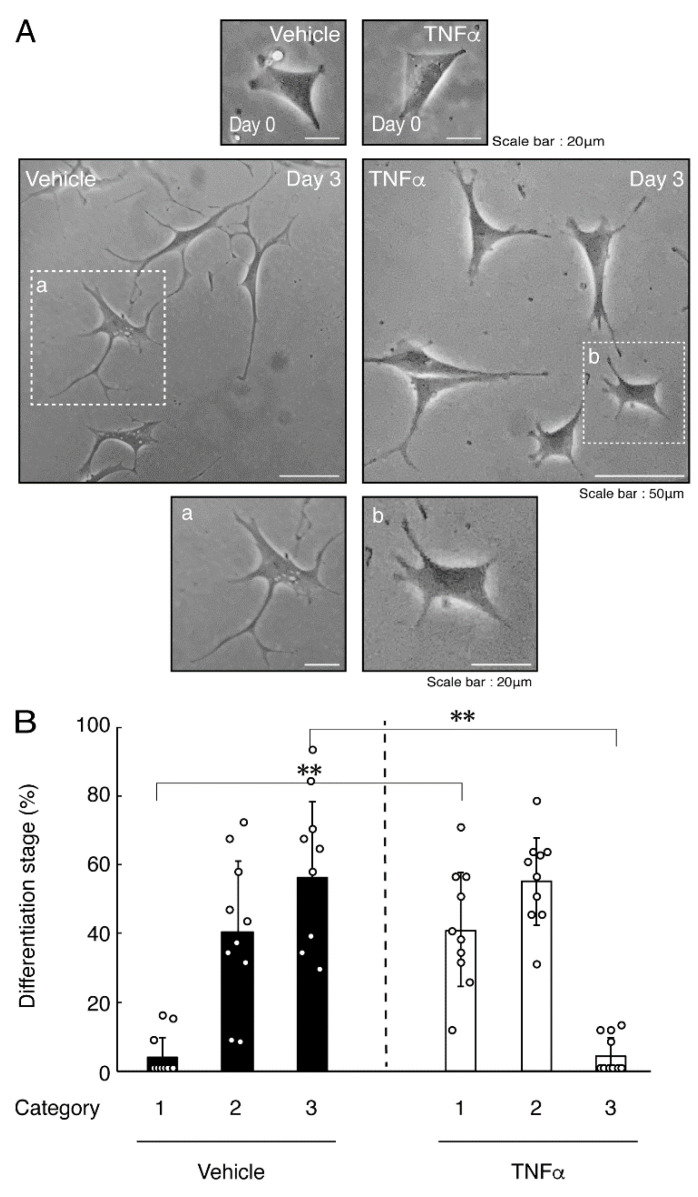
Treatment of cells with TNFα inhibits oligodendroglial cell morphological differentiation. (**A**,**B**) FBD-102b cells were treated with TNFα or control vehicles and were allowed to be differentiated for 0 or 3 days. Lower panels a and b are magnified dotted squares a and b of middle panels. Differentiation efficiencies were divided into 3 categories (cells with primary processes were classified as category 1; cells with secondary processes branched from primary processes were classified as category 2; and cells with third processes branched from secondary processes or with widespread membranes were classified as category 3) and depicted in graphs (**, *p* < 0.01; n = 10 [taking one picture each from 10 independent experiments]).

**Figure 2:**
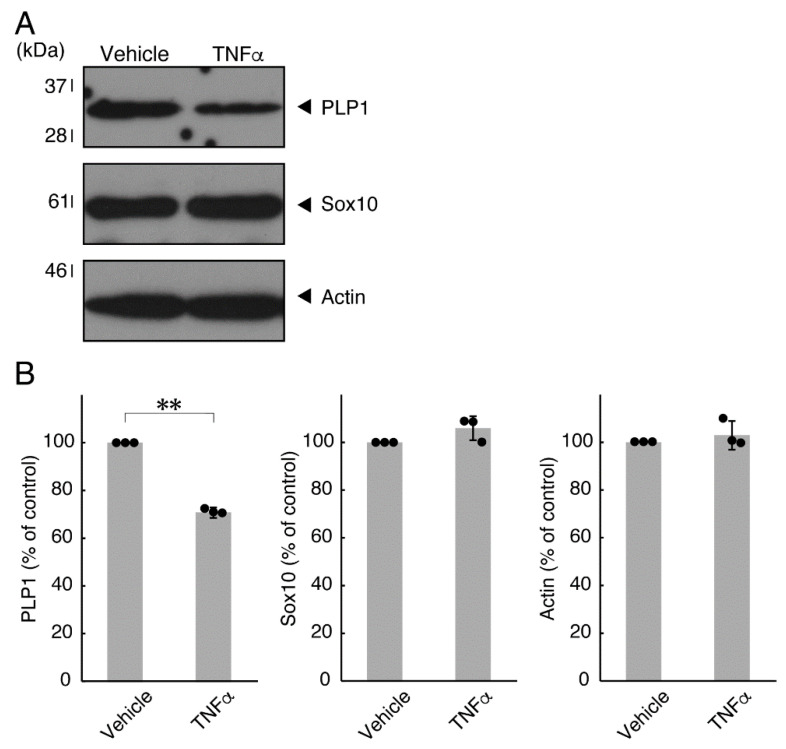
Effects of TNFα on oligodendroglial cell marker protein expression. (**A**,**B**) Cells were treated with TNFα or control vehicles. The PLP1, Sox10, or control actin blot is shown. Immunoreactive band intensities were also compared to be depicted in graphs (**, *p* < 0.01; n = 3 blots [obtaining one sample each from 3 independent experiments]).

In order to investigate whether TNFα has a direct or indirect effect on the morphology of oligodendroglial cells, we treated FBD-102b cells with TNFα. Following the induction of differentiation, cells, which were treated with control vehicles, exhibited differentiated phenotypes with multiple branches from cell bodies and/or secondary and third branches from primary and secondary branches, respectively. In contrast, treatment with TNFα resulted in inhibiting differentiation. Especially, TNFα increased the number of cells with primary branches whereas decreased that of cells with third branches (Figure 1A,B). These results were supported by decreased expression levels of oligodendroglial cell differentiation and myelin marker proteolipid protein 1 (PLP1). PLP1 is known to be the specific marker of the terminal differentiated oligodendroglial cells in the CNS and is essential for oligodendroglial cell differentiation and in turn myelination [1,2,3,4]. In addition, oligodendrocyte lineage marker Sox10 as well as control actin proteins were comparable in cells treated with control vehicles and TNFα (Figure 2A,B).

**Figure 3:**
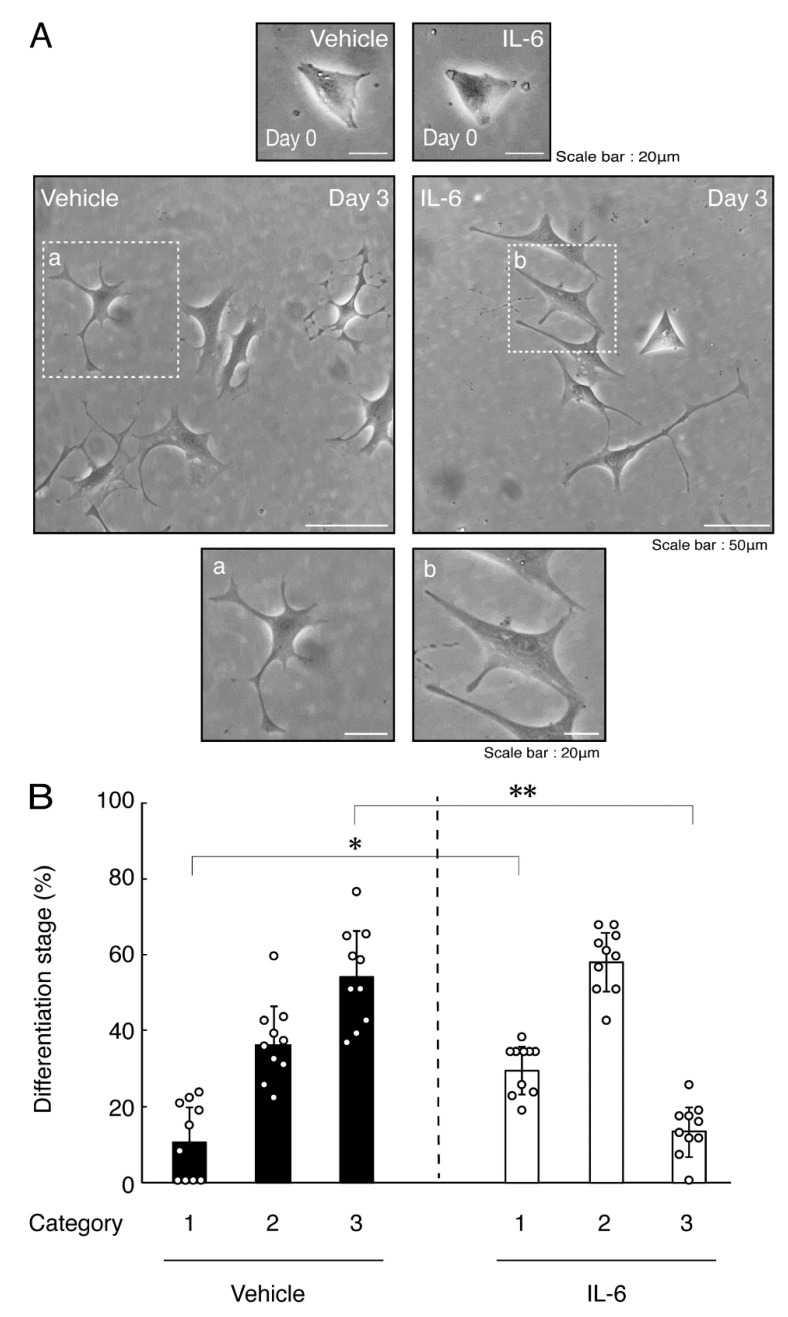
Treatment of cells with IL-6 inhibits oligodendroglial cell morphological differentiation. (**A**,**B**) FBD-102b cells were treated with IL-6 or control vehicles and were allowed to be differentiated. Lower panels a and b are magnified dotted squares a and b of middle panels. Differentiation efficiencies were divided into 3 categories and depicted in graphs (**, *p* < 0.01; *, *p* < 0.05; n = 10 [taking one picture each from 10 independent experiments]).

**Figure 4:**
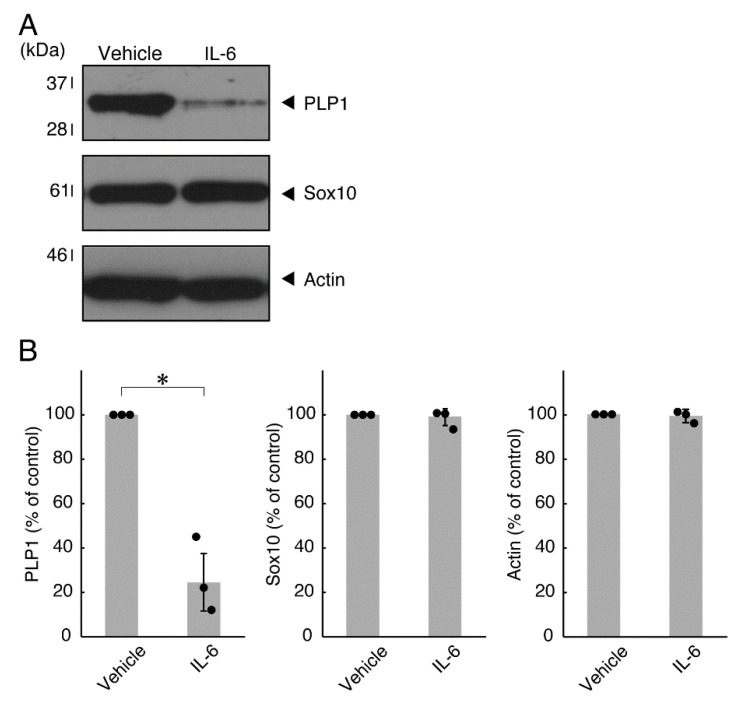
Effects of IL-6 on oligodendroglial cell marker protein expression. (**A**,**B**) Cells were treated with IL-6 or control vehicles. The PLP1, Sox10, or control actin blot is shown. Immunoreactive band intensities were also compared to be depicted in graphs (*, *p* < 0.05; n = 3 blots [obtaining one sample each from 3 independent experiments]).

Next, we explored whether IL-6 also has an effect on the morphology of oligodendroglial cells. Treatment with IL-6 but not with control vehicles resulted in inhibiting differentiation (Figure 3A,B), consistent with the results of decreased expression levels of PLP1. In contrast, Sox10 and actin proteins were comparable in cells treated with control vehicles and IL-6 (Figure 4A,B). In comparison, IL-11 of the IL-6 family member proteins weakly but significantly promoted morphological differentiation (Figure S1) with increased levels of marker proteins (Figure S2).

**Figure 5:**
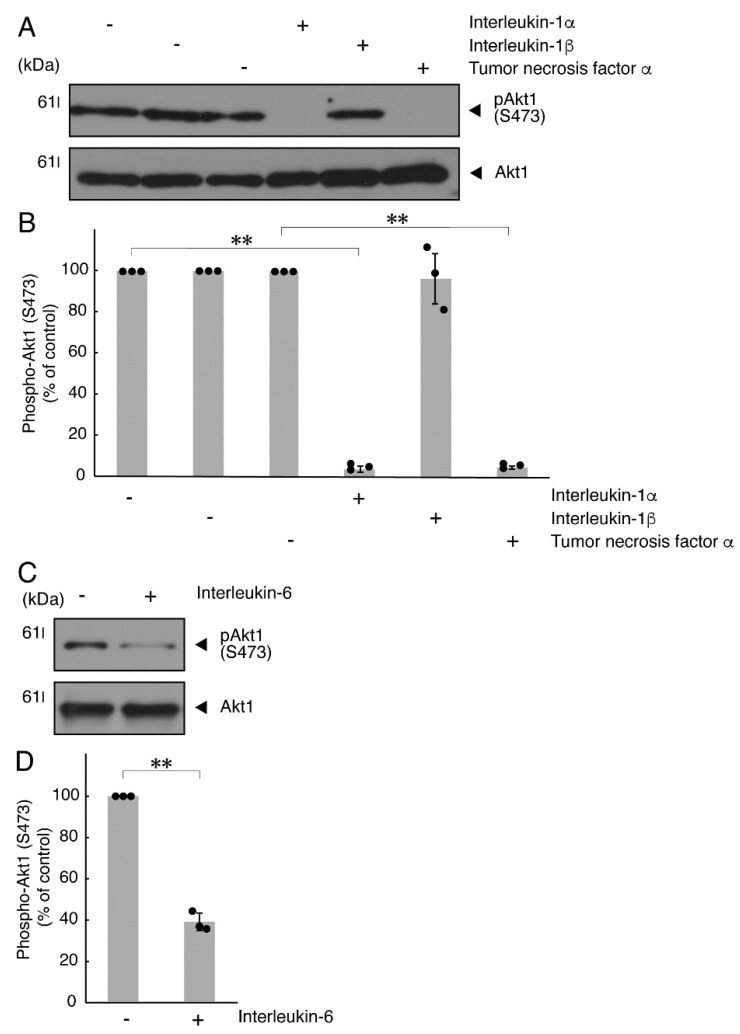
TNFα or IL-6 inhibits phosphorylation of Akt kinase. (**A**,**B**) Cells were treated with control vehicles (-), interleukin-1α (IL-1α as a positive control)/interleukin-1α (IL-1β as a negative control) or tumor necrosis factor α (TNFα). The (pS473)Akt1 or Akt1 blot is shown. Immunoreactive band intensities for (pS473)Akt1 were also compared to be depicted in graphs (**, *p* < 0.01; n = 3 blots [obtaining one sample each from 3 independent experiments]). (**C**,**D**) Cells were treated with control vehicles (-) or interleukin-6 (IL-6). The (pS473)Akt1 or Akt1 blot is shown. Immunoreactive band intensities for (pS473)Akt1 were also compared to be depicted in graphs (**, *p* < 0.01; n = 3 blots [obtaining one sample each from 3 independent experiments]). The vertical values in graphs were evaluated as (pS473)Akt1/total Akt1.

It is well known that oligodendroglial cell differentiation and myelination are strictly related to Akt kinase whose phosphorylation at the 473 position of serine is correlated with the activity [5,6]. Following the induction of differentiation, Akt kinase was phosphorylated. In contrast, treatment with TNFα inhibited the phosphorylation levels of Akt kinase (Figure 5A,B). Similar results were observed in the cases of a prototypic inflammatory cytokine IL-1α (Figure 5A,B) and IL-6 (Figure 5C,D) but not of another inflammatory cytokine IL-1β (Figure 5A,B). In contrast, IL-11 weakly but significantly promoted Akt kinase phosphorylation (Figure S3). Taken together with the results described above, TNFα or IL-6 has the ability to inhibit oligodendroglial cell morphological differentiation with decreased levels of PLP1 expression and Akt kinase phosphorylation.

### 3.2. Hesperetin Recovers TNFα- or IL-6-Mediated Inhibition of Differentiation Whose State Is Related to Akt Phosphorylation

**Figure 6:**
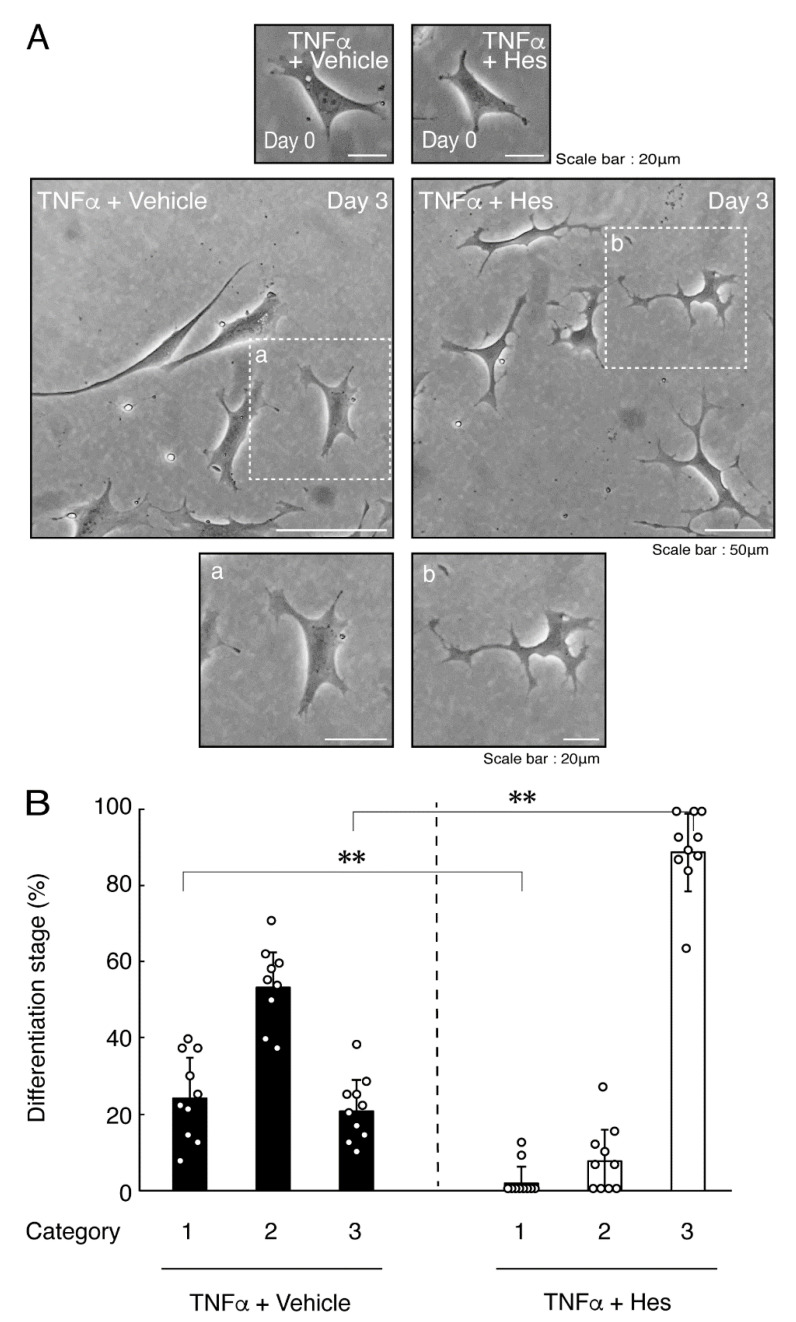
Hesperetin recovers TNFα inhibition of oligodendroglial cell morphological differentiation. (**A**,**B**) Differentiation of FBD-102b cells treated with TNFα was induced in the presence of hesperetin (Hes) or control vehicles. Lower panels a and b are magnified dotted squares a and b of middle panels. Differentiation efficiencies were divided into 3 categories and depicted in graphs (**, *p* < 0.01; n = 10 [taking one picture each from 10 independent experiments]).

**Figure 7:**
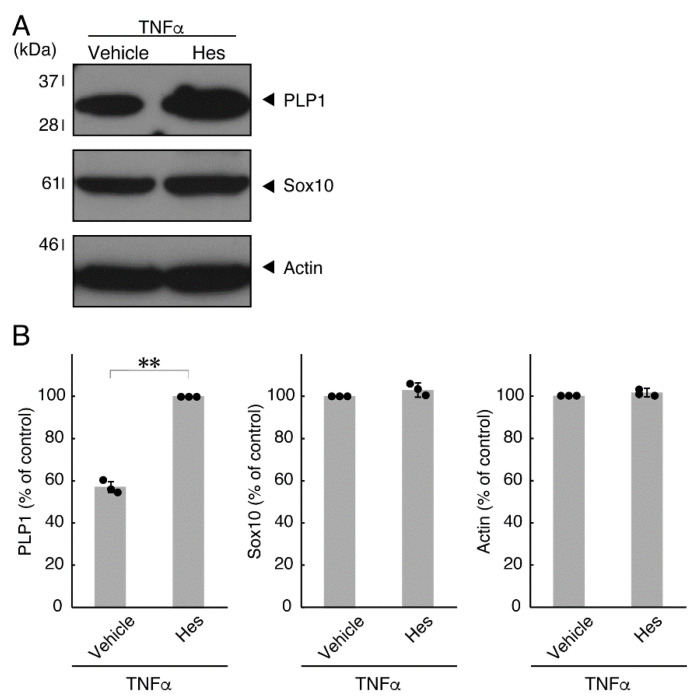
Effects of hesperetin on expression levels of oligodendroglial cell marker proteins in the presence of TNFα. (**A**,**B**) Cells were treated with TNFα in the presence of hesperetin (Hes) or control vehicles. The PLP1, Sox10, or control actin blot is shown. Immunoreactive band intensities were also compared to be depicted in graphs (**, *p* < 0.01; n = 3 blots [obtaining one sample each from 3 independent experiments]).

**Figure 8:**
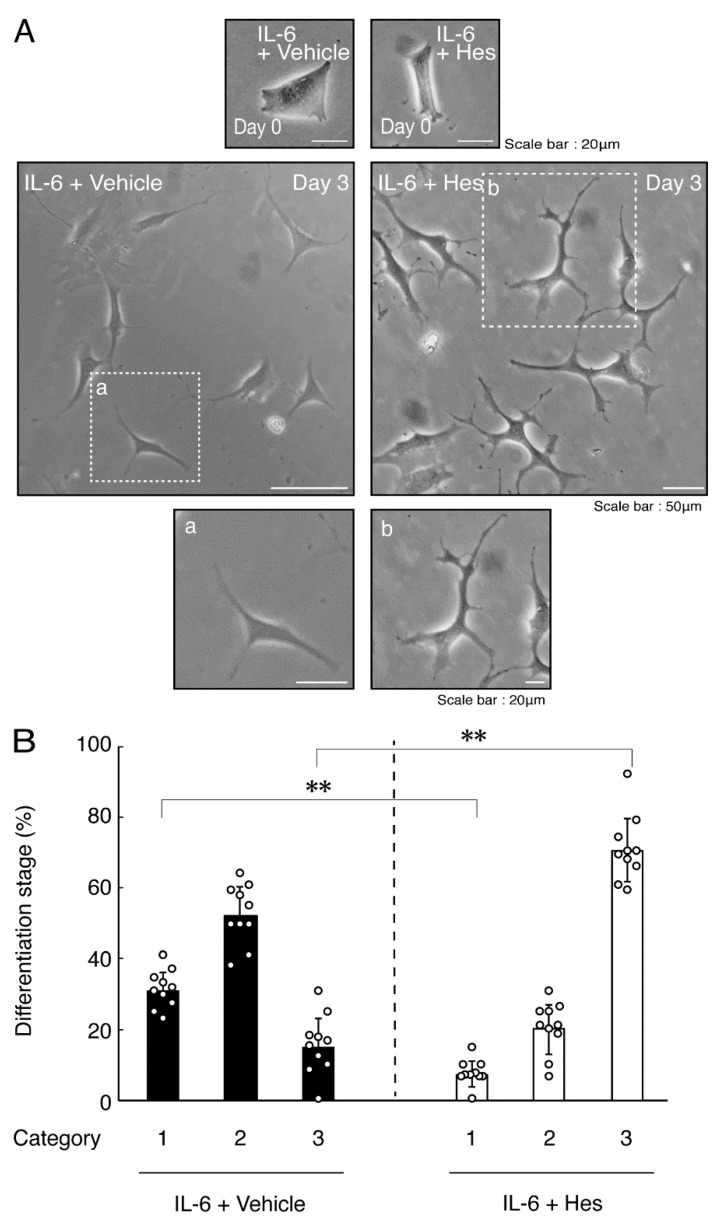
Hesperetin recovers IL-6 inhibition of oligodendroglial cell morphological differentiation. (**A**,**B**) Differentiation of FBD-102b cells treated with IL-6 was induced in the presence of hesperetin (Hes) or control vehicles. Lower panels a and b are magnified dotted squares a and b of middle panels. Differentiation efficiencies were divided into 3 categories and depicted in graphs (**, *p* < 0.01; n = 10 [taking one picture each from 10 independent experiments]).

**Figure 9:**
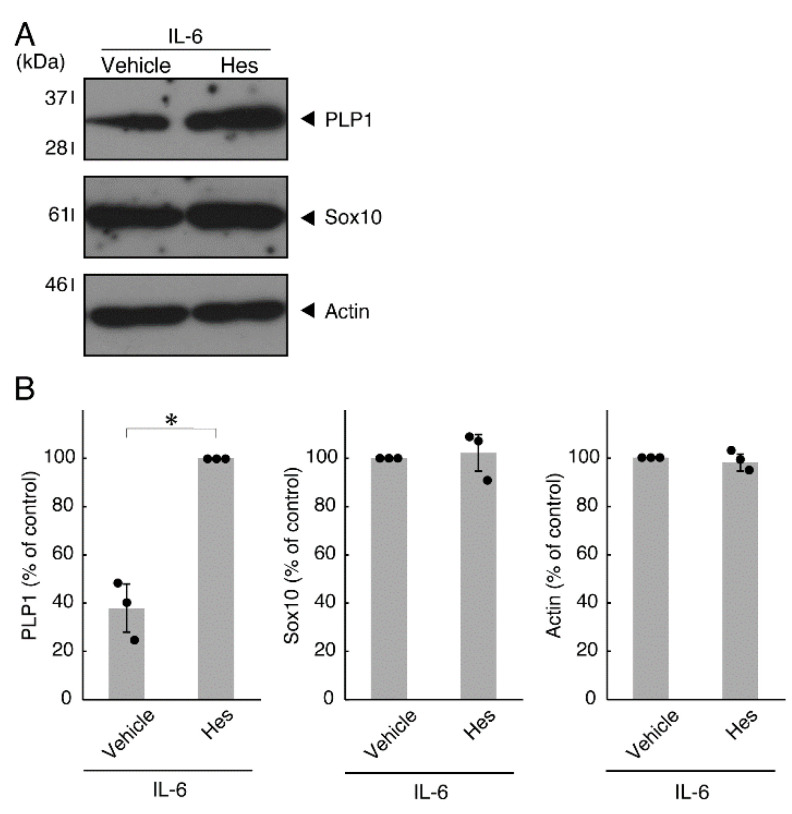
Effects of hesperetin on expression levels of oligodendroglial cell marker proteins in the presence of IL-6. (**A**,**B**) Cells treated with IL-6 in the presence of hesperetin (Hes) or control vehicles. The PLP1, Sox10, or control actin blot is shown. Immunoreactive band intensities were also compared to be depicted in graphs (*, *p* < 0.05; n = 3 blots [obtaining one sample each from 3 independent experiments]).

**Figure 10:**
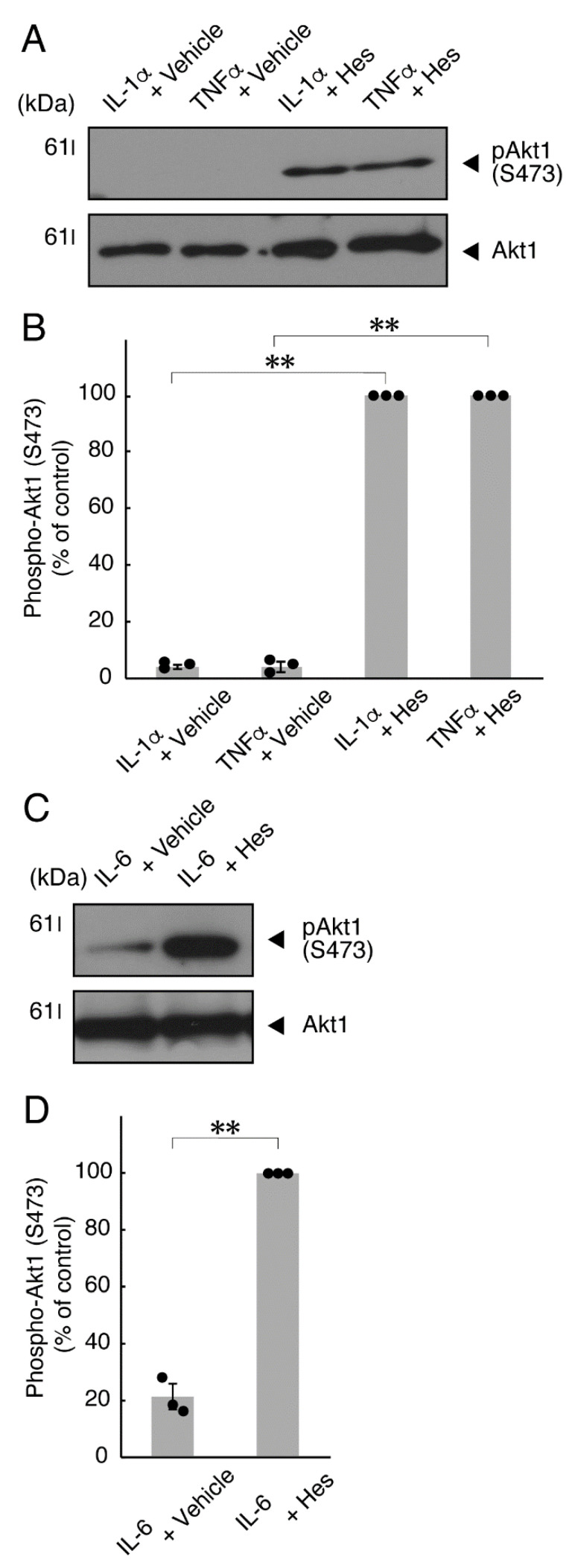
Hesperetin recovers inhibition of Akt kinase phosphorylation by TNFα or IL-6. (**A**,**B**) Cells were treated with TNFα or IL-1α in the presence of control vehicles or hesperetin (Hes). The (pS473)Akt1 or Akt1 blot is shown. Immunoreactive band intensities for (pS473)Akt1 were also compared to be depicted in graphs (**, *p* < 0.01; n = 3 blots [obtaining one sample each from 3 independent experiments]). (**C**,**D**) Cells were treated with IL-6 in the presence of control vehicles or hesperetin. The (pS473)Akt1 or Akt1 blot is shown. Immunoreactive band intensities for (pS473)Akt1 were also compared to be depicted in graphs (**, *p* < 0.01; n = 3 blots [obtaining one sample each from 3 independent experiments]). The vertical values in graphs were evaluated as (pS473)Akt1/total Akt1.

Hesperetin is an aglycone of hesperidin, which is a flavonoid abundant in the pericarp of citrus fruits. Hesperetin has been known to blunt progressing neurological disorders such as Alzheimer’s disease potentially by inhibiting their anti-inflammatory effects [15,16]. Despite the known effects of hesperetin on neuronal cells [15,16], it has remained unclear whether it affects oligodendroglial cells. We thus sought to test the effects of hesperetin on FBD-102b cells. Hesperetin actually promoted morphological differentiation in a dose-dependent manner but treatment of cells with hesperetin at a concentration of 10 μM was sufficient to induce it (Figure S4). Next, we tested whether hesperetin recovers TNFα-mediated inhibition of oligodendroglial cell morphological differentiation. Hesperetin resulted in recovering the inhibitory morphological differentiation although the control vehicles did not have significant effects. Especially, hesperetin decreased the number of cells with primary branches whereas increased that of cells with third branches (Figure 6A,B). These data were consistent with the results that the expression levels of PLP1 were increased by treatment with hesperetin. In contrast, Sox10 and actin proteins were comparable in the presence and absence of hesperetin (Figure 7A,B). Similar effects of hesperetin on cell morphologies and marker protein expression levels were obtained in the case of IL-6 (Figure 8A,B and Figure 9A,B). Phosphorylation levels of Akt kinase inhibited by TNFα were also recovered by treatment with hesperetin, consistent with the results on its effect for prototypic inflammatory cytokine IL-1α (Figure 10A,B). Similar effects were observed in the case of IL-6 (Figure 10C,D). Taken together with the results described above, hesperetin recovers TNFα or IL-6 inhibition of oligodendroglial cell morphological differentiation as well as levels of PLP1 expression and Akt kinase phosphorylation.

## 4. Discussion

Inflammatory cytokines TNFα and IL-6 play key roles in oligodendroglial cell and myelin diseases such as MS [7,8,9,10]. In the present study, we report that treatment with TNFα or IL-6 in FBD-102b cells directly inhibits morphological differentiation. Each effect of TNFα or IL-6 on cells was supported by the decreased expression levels of differentiation and myelin marker proteins. Importantly, the levels of phosphorylation of Akt kinase, whose states are strictly related to oligodendroglial cell differentiation and myelination [5,6], were also decreased. It is of note that hesperetin, a citrus flavonoid, recovers TNFα- or IL-6-mediated inhibition of morphological differentiation. These results were also supported by the increased levels of differentiation and myelin marker protein expression and of Akt kinase phosphorylation. Hesperetin may be a potential chemical drug candidate to recover inflammatory cytokine-mediated inhibition of oligodendroglial cell differentiation.

One of the cytokines that has major effects on MS and other inflammatory autoimmune diseases is TNFα. The immunoreactivities of TNFα, particularly in pathological tissues, have been identified as antigens expressed in multiple cell types, such as macrophages, dendritic cells, natural killer T cells, neutrophils, T and B cells, as well as some nonhematopoietic cells [17]. Increasing evidence also indicates that TNFα is involved in the progression of the pathology of MS. MS patients display increased levels of TNFα at the sites of active pathological lesions. In addition, the levels of TNFα are increased in the cerebrospinal fluid and serum of some patients [18,19]. The increased levels of TNFα are correlated with the severity of active lesions in the progression of MS [17,18,19]. TNFα itself may lead to the inhibition of possible remyelination as well as differentiation and myelination in oligodendroglial cells during pathological processes. Indeed, treatment with TNFα in nerve tissues strongly inhibits MBP expression and decreases the number of MBP-positive cells [20]. On the other hand, one of the major receptors for TNFα is known to be TNF receptor superfamily member 1A (TNFR1A), which contains a single death domain. Another major receptor for TNFα is TNF receptor superfamily member 2 (TNFR2), which generally has cellular protective effects without a death domain [21,22]. It is unclear which TNFα receptor, TNFR1A or TNFR2, is involved in the inhibition of oligodendroglial cell morphological differentiation, but TNFR1A appears to have abilities that neither promote growth and the subsequent differentiation in oligodendroglial cell precursor cells nor accelerate remyelination [23,24]. To date, it has remained unknown whether TNFR1A is the target of hesperetin to inhibit oligodendroglial cell morphological differentiation. Further studies are required to examine the relationship between TNFR1A and hesperetin.

Another major cytokine that has particular effects in MS and other inflammatory autoimmune diseases is IL-6, but not IL-11, of the IL-6 family cytokine proteins. IL-6 is also secreted from multiple cell types, including nonhematopoietic cells. As with TNFα, it is suggested that IL-6 secretion is related to MS and other inflammatory autoimmune diseases [25,26,27,28,29,30]. Actually, a chimeric derivative of IL-6 and soluble IL-6 receptor (IL6RIL6 chimera) was demonstrated to prevent not only oligodendroglial cell degradation and probable myelin degradation but also neuronal cell degradation under experimental demyelinating, pathological conditions in organotypic hippocampal slices [31,32], although it remains unclear whether and how hesperetin binds to have an effect on IL-6 receptor.

Flavonoids, including hesperetin, are known to decrease the neuroinflammation involved in the progression of neurodegenerative disorders such as Alzheimer’s disease, Parkinson’s disease, and amyotrophic lateral sclerosis as well as MS, but not to directly inhibit the degeneration of neuronal and glial cells [33,34]. Specifically, it has been reported that flavonoids block the secretion of inflammatory cytokines such as TNFα and IL-6 from microglial cells and delay the progression of neurodegenerative disorders [33,34]. It has also been reported that they act as antioxidant molecules to reduce the levels of oxidative stress through reactive oxygen species (ROSs)-scavenging mechanisms as antioxidant molecules [35,36]. Additionally, increasing evidence indicates that hesperetin binds to some mammalian species proteins or proteins such as the S1 spike proteins of severe acute respiratory syndrome coronavirus 2 (SARS-CoV-2) [37]. Among the attractive hesperetin target and binding proteins, protein tyrosine phosphatase 1B (PTP1B) is of particular note [38]. Since PTP1B is known to be a negative regulator belonging to insulin receptor substrate 1 (IRS1) and Akt kinase phosphorylating signaling [39,40], inhibition of PTP1B as the hesperetin target likely leads to upregulation of signaling through Akt kinase, reminding us that hesperetin can recover TNFα- or IL-6-mediated inhibition of oligodendroglial cell morphological differentiation by upregulating Akt kinase as the major key signaling molecule underlying oligodendroglial cell differentiation and myelination [5,6]. However, it has remained unclear whether hesperetin directly inhibits the activities of PTP1B itself by binding to PTP1B.

Here, for the first time, we show that inflammatory cytokine TNFα or IL-6 directly affects the inhibition of oligodendroglial cell morphological differentiation. Their inhibition was related to changes in the phosphorylation of Akt kinase. Treatment with hesperetin recovered the inhibitory effects, illustrating its potential as a chemical drug for inflammatory cytokine-related oligodendroglial cell diseases. Further studies are needed to promote our understanding not only of how TNFα or IL-6 has direct effects on inhibiting oligodendroglial cell morphological differentiation and, in turn, possible myelination through mediating Akt kinase activities in coculture and model animal levels, but also of whether and how other inflammatory cytokines inhibit morphological differentiation and myelination. Additional studies are needed to identify intracellular or extracellular proteins or other bioorganic target(s) of hesperetin that recover the effects of inflammatory cytokines. These studies will enable the development of therapeutic drugs for inflammatory cytokine-related oligodendroglial cell and myelin diseases.

## Data Availability

Not applicable.

## Supplementary Materials

The following supporting information can be downloaded at: https://www.mdpi.com/article/10.3390/neurolint14020039/s1. Figure S1: Treatment of cells with IL-11 weakly promotes oligodendroglial cell morphological differentiation. Figure S2: Effects of IL-11 on oligodendroglial cell marker protein expression. Figure S3: IL-11 weakly promotes phosphorylation of Akt kinase. Figure S4: Effects of hesperetin on oligodendroglial cell morphological differentiation. Figure S5: Original size gel of immunoblots.
