# Supplementary material for: Hesperetin, a Citrus Flavonoid, Ameliorates Inflammatory Cytokine-Mediated Inhibition of Oligodendroglial Cell Morphological Differentiation
Source: Neurol Int. 2022 May 31;14(2):471–87. doi: 10.3390/neurolint14020039 (PMC9230394; doi:10.3390/neurolint14020039)
Supplement: Supplementary file 1 [file neurolint-14-00039-s001.zip › neurolint-1735540-supplementary.pdf]

Supplemental figure legends

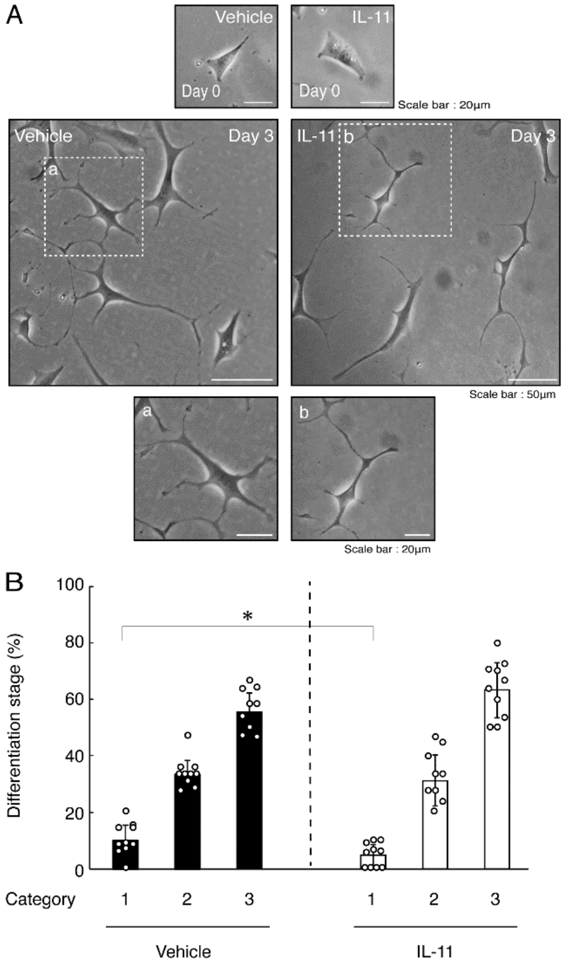

Figure S1. Treatment of cells with IL-11 weakly promotes oligodendroglial cell morphological differentiation. (A, B) FBD-102b cells were treated with IL-11 or control vehicles and were allowed to be differentiated for 0 or 3 days. Lower panels a and b are magnified dotted squares a and b of middle panels. Differentiation efficiencies were divided into 3 categories and depicted in graphs (*, $p<0.05$; n = 10 [taking one picture each from 10 independent experiments]).

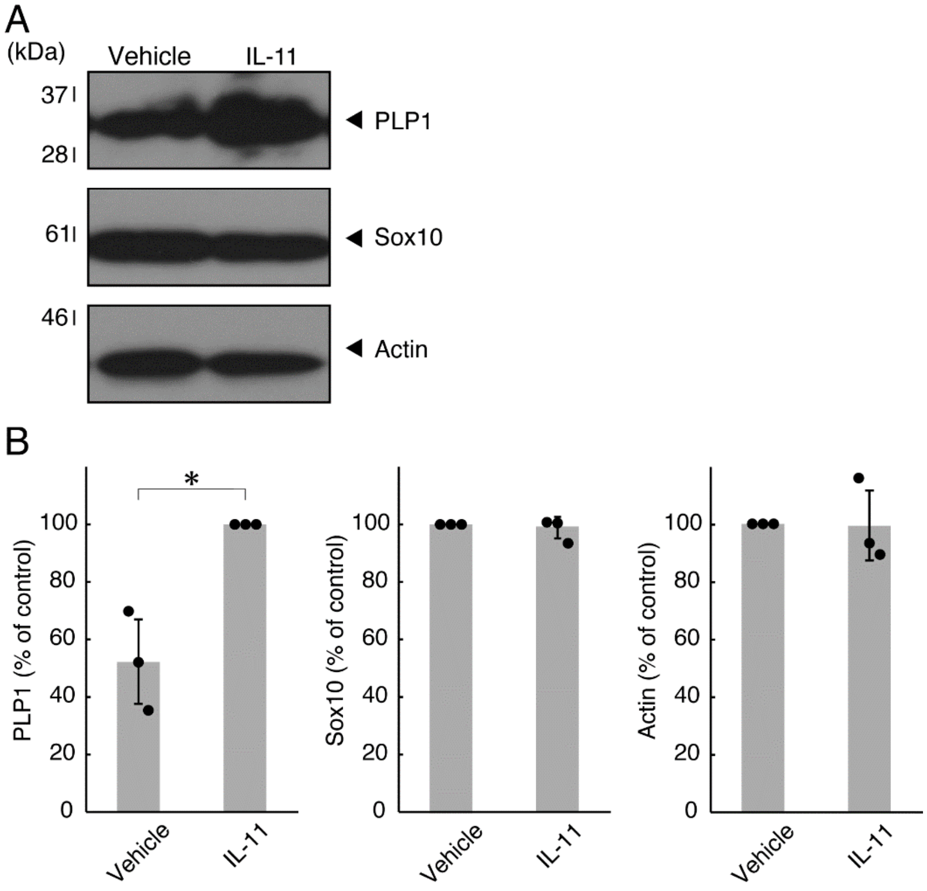

Figure S2. Effects of IL-11 on oligodendroglial cell marker protein expression. (A, B) Cells were treated with IL-11 or control vehicles. The PLP1, Sox10, or control actin blot is shown. Immunoreactive band intensities were also compared to be depicted in graphs (*, p<0.05; n = 3 blots [obtaining one sample each form 3 independent experiments]).

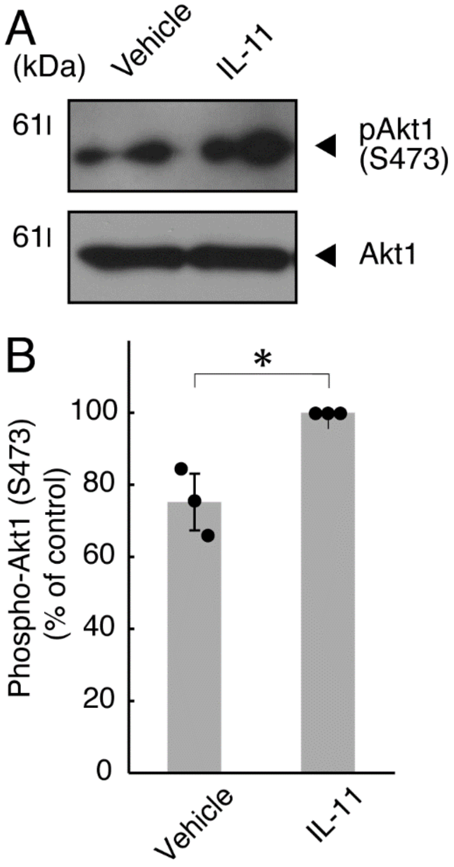

Figure S3. IL-11 weakly promotes phosphorylation of Akt kinase. (A, B) Cells were treated with control vehicles or IL-11. The (pS473)Akt1 or Akt1 blot is shown. Immunoreactive band intensities for (pS473)Akt1 were also compared to be depicted in graphs (*, p<0.05; n = 3 blots [obtaining one sample each form 3 independent experiments]). The vertical value in a graph was evaluated as (pS473)Akt1/total Akt1.

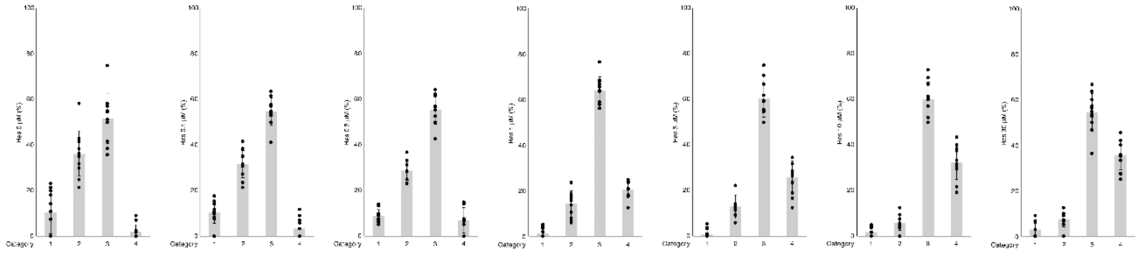

Figure S4. Effects of hesperetin on oligodendroglial cell morphological differentiation. FBD-102b cells were allowed to be differentiated 3 days in the presence

of 0 (control vehicles) to 30 μM of hesperetin (Hes). Differentiation efficiencies were divided into 4 categories (cells with primary processes were classified as the category 1; cells with secondary processes branched from primary processes were classified as the category 2; cells with third processes branched from secondary processes were classified as the category 3; and cells with widespread membranes were classified as the category 4) and depicted in graphs (n = 10 fields).

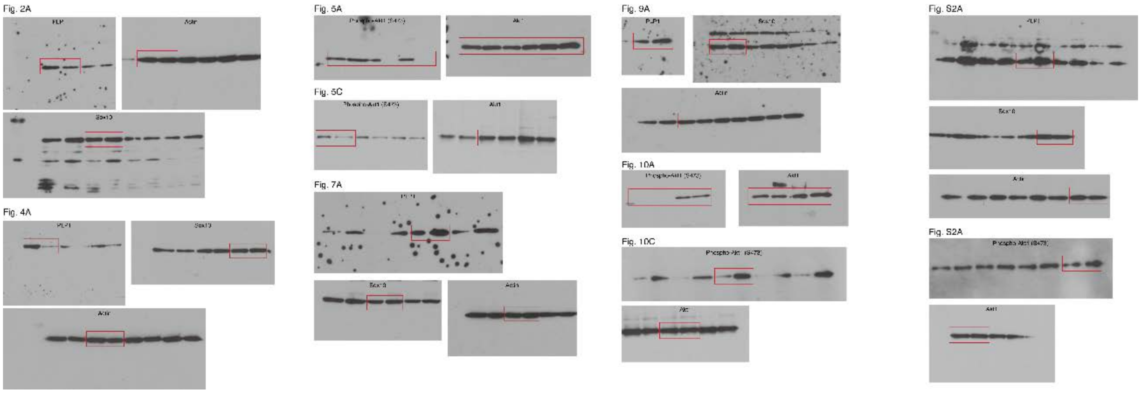

Figure S5. Original size gel of immunoblots. Used portions in figures are surrounded by red squares.
